# The venom composition of the parasitic wasp *Chelonus inanitus *resolved by combined expressed sequence tags analysis and proteomic approach

**DOI:** 10.1186/1471-2164-11-693

**Published:** 2010-12-07

**Authors:** Bruno Vincent, Martha Kaeslin, Thomas Roth, Manfred Heller, Julie Poulain, François Cousserans, Johann Schaller, Marylène Poirié, Beatrice Lanzrein, Jean-Michel Drezen, Sébastien JM Moreau

**Affiliations:** 1 UMR 6035 CNRS, Institut de Recherche sur la Biologie de l'Insecte, Faculté des Sciences et Techniques, Université François-Rabelais, Parc Grandmont, 37200 Tours, France; 2 Institute of Cell Biology, University of Berne, Baltzerstrasse 4, CH-3012 Berne, Switzerland; 3 Department of Clinical Research, University of Bern, Murtenstrasse 35, CH-3010 Berne, Switzerland; 4 CEA, DSV, Institut de Génomique, Genoscope, 2 rue Gaston Crémieux, CP5706, 91057 Evry, France; 5 UMR INRA-UM2 1231, Laboratoire Biologie Intégrative et Virologie des Insectes, Université de Montpellier 2, Place E. Bataillon, CC54, 34095 Montpellier cedex 05, France; 6 Department of Chemistry and Biochemistry, University of Berne, Freiestrasse 3, CH-3012 Berne, Switzerland; 7 UMR INRA (1301)-CNRS (6243)-Université Nice Sophia Antipolis, "Interactions Biotiques et Santé Végétale", Institut Agrobiotech, 400 Route des Chappes, 06903 Sophia Antipolis, France

## Abstract

**Background:**

Parasitic wasps constitute one of the largest group of venomous animals. Although some physiological effects of their venoms are well documented, relatively little is known at the molecular level on the protein composition of these secretions. To identify the majority of the venom proteins of the endoparasitoid wasp *Chelonus inanitus *(Hymenoptera: Braconidae), we have randomly sequenced 2111 expressed sequence tags (ESTs) from a cDNA library of venom gland. In parallel, proteins from pure venom were separated by gel electrophoresis and individually submitted to a nano-LC-MS/MS analysis allowing comparison of peptides and ESTs sequences.

**Results:**

About 60% of sequenced ESTs encoded proteins whose presence in venom was attested by mass spectrometry. Most of the remaining ESTs corresponded to gene products likely involved in the transcriptional and translational machinery of venom gland cells. In addition, a small number of transcripts were found to encode proteins that share sequence similarity with well-known venom constituents of social hymenopteran species, such as hyaluronidase-like proteins and an Allergen-5 protein.

An overall number of 29 venom proteins could be identified through the combination of ESTs sequencing and proteomic analyses. The most highly redundant set of ESTs encoded a protein that shared sequence similarity with a venom protein of unknown function potentially specific of the *Chelonus *lineage. Venom components specific to *C. inanitus *included a C-type lectin domain containing protein, a chemosensory protein-like protein, a protein related to yellow-e3 and ten new proteins which shared no significant sequence similarity with known sequences. In addition, several venom proteins potentially able to interact with chitin were also identified including a chitinase, an imaginal disc growth factor-like protein and two putative mucin-like peritrophins.

**Conclusions:**

The use of the combined approaches has allowed to discriminate between cellular and truly venom proteins. The venom of *C. inanitus *appears as a mixture of conserved venom components and of potentially lineage-specific proteins. These new molecular data enrich our knowledge on parasitoid venoms and more generally, might contribute to a better understanding of the evolution and functional diversity of venom proteins within Hymenoptera.

## Background

Hymenopteran venoms have been intensively studied in social species such as bees, bumblebees, wasps, hornets and ants [1-6]. Most of the major allergens have been identified in species of medical importance through a combination of transcriptomic, proteomic, peptidomic and glycomic techniques recently gathered under the newly proposed term of venomic approaches [7]. In comparison, little has been done on the venom composition of parasitoid Hymenoptera although they represent more than 75% of described hymenopteran species and 10-20% of all insect species [8]. Fundamental benefits expected from venomic approaches applied to parasitic wasp venoms would consist, for example, in the discrimination between cellular transcripts present in the venom glands and those encoding true venom proteins, through the proteomic analysis of venom fluid. Moreover comprehensive analyses would allow a deeper characterization of weakly expressed venom components and a comparative work aiming at retracing the evolutionary history of hymenopteran venoms. Parasitoid venom proteins also constitute an underestimated source of toxins that could be studied for a variety of applied uses.

Parasitic wasps constitute by far the largest group of parasitic insects with an estimated total number of species of approximately 250 000 [9]. Some develop outside (ectoparasitoids) and others inside (endoparasitoids) the body of an insect or other arthropod host and, depending on the species, various stages of the host can be parasitized (egg, egg-larval, larval, pupal and adult parasitoids). In ectoparasitoid species, venoms often induce paralysis and/or regulate host development, metabolism and immune responses [10-12]. Venom proteins from endoparasitic wasps are predominately involved in regulation of host physiology and immune responses alone or in combination with other factors of maternal origin such as polydnaviruses (PDVs) or virus-like particles present in the venom itself or produced in the ovaries and ovarian fluids [13-17]. For example, venoms can synergize the effects of PDVs [18,19] and can interfere with host's humoral [20-22] and cellular immune components [23-26].

To date, less than 50 proteins have been individually identified and characterized from the venoms of a restricted number of parasitoid wasps species [15,27]. Broader studies have also previously investigated the composition of parasitoid venoms by the separate use of proteomic or transcriptomic approaches combined with bioinformatic analyses. A recent analysis of the venom proteome of the pupal ectoparasitoid wasp *Nasonia vitripennis *has been published, that benefited from the sequencing and annotation of this wasp genome [28]. Twelve venom proteins from the endoparasitoid *Pteromalus puparum *were also identified recently using a proteomic approach [29]. On the other hand, transcriptome analyses allowed the identification of venom proteins in the pupal endoparasitoid *Pimpla hypochondriaca *[30-32] and in two adult endoparasitoid species of the genus *Microctonus *[33]. Although these works are undoubtedly of great interest, most of them did not provide absolute evidence that all identified proteins were venom components. Therefore, there is still a crucial need for extensive analyses by combining various techniques of investigation at the molecular level to allow comparisons between species.

*Chelonus inanitus *(Hymenoptera: Braconidae) is original among the parasitoid species currently studied in being an egg-larval endoparasitoid species. Indeed it oviposits into the eggs of its host, *Spodoptera littoralis *(Lepidoptera: Noctuidae) and the parasitoid larva then develops inside the host embryo and early larval stages. Due to its lifestyle, *C. inanitus *must thus face up to particular physiological constraints imposed by its immature hosts. The venom, along with PDVs produced in the reproductive system of this wasp, are essential for successful parasitism as they protect the parasitoid from encapsulation by host's immune cells [34], interfere with the host's nutritional physiology [35] and induce a developmental arrest in the prepupal stage [18,36,37]. Venom of *C*. *inanitus *by itself alters the membrane permeability of host hemocytes, has a transient paralytic effect [38] and synergizes the effect of the PDVs on host development [18]. The data gathered on the functions of its venom make *C. inanitus *a valuable model for investigating the venom proteins. At least 25 proteins were found [38] but their sequences were unknown. The sequences of only two venom proteins from another species of the subfamily Cheloninae, *Chelonus *sp. near *curvimaculatus*, had been described to date [39,40].

We report here the analysis of *C. inanitus *venom gland products based on the sequencing of clones of a cDNA library and on mass spectrometry analysis of venom proteins. This is the first time that this combination of techniques was applied to identify venom proteins from an endoparasitic wasp. The data obtained might contribute to acquiring a more comprehensive view on the origin and functional diversity of venom proteins among Hymenoptera.

## Results and Discussion

### General overview of the cDNA library of *C. inanitus *venom gland

**Figure 1:**
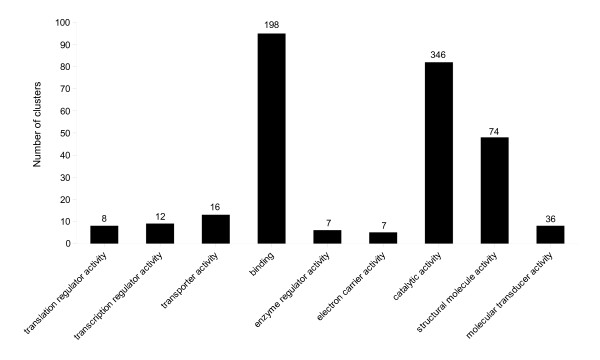
**Functional characterization of assembled clusters from *C. inanitus *venom gland**. Histograms show the distribution of sequence clusters from *C. inanitus *venom gland transcriptome according to the 9 molecular functional categories at level 2 of the gene ontology system. Values figuring at the top of bars indicate the respective number of ESTs classified into each functional category.

The 2111 ESTs from the venom glands of *C. inanitus *were clustered into 488 clusters (95 contigs and 393 singletons, Table 1). The number of ESTs in each contig ranged from 2 to 534 and these clusters were considered as putative unigenes. The deduced sequences from 250 clusters (56 contigs and 194 singletons, 56.85% of all ESTs) shared significant similarities with protein sequences deposited in non-redundant databases (EMBL/Genbank), a proportion comparable to that found by Crawford *et al*. [33] which have studied the venom gland transcriptome of the parasitoid wasp *Microctonus hyperodae*. Of these products, 164 (corresponding to 36 contigs and 128 singletons, 21.5% of all ESTs) shared significant similarity with proteins with assigned molecular functions in the gene ontology database. This relatively low percentage is explained in part by the fact that the function of the most represented sequence (534 ESTs) referred below as Ci-23a, is unknown. At level 2 of the gene ontology system, clusters were classified into 9 molecular functional categories (Figure 1), among which "binding" (GO:0005488) and "catalytic activity" (GO:0003824) categories were over-represented (95 and 82 clusters respectively). Interestingly, these categories were also the most common functional categories assigned to the venom glands ESTs from the saw-scaled viper, *Echis ocellatus *[41] and from the solitary hunting wasp species, *Orancistrocerus drewseni *[42]. Catalytic activity and binding categories thus constitute a hallmark of the venom gland transcriptomes analysed to date. A "structural molecule activity" function (GO:0005198) has been assigned to 48 clusters (20 contigs, 28 singletons) that corresponded essentially to genes coding for structural constituents of ribosomes (17 contigs, 21 singletons). In addition, products of genes functionally annotated as "translation regulator activity" (GO:0045182) (8 singletons) and "transcription regulator activity" (GO:0030528) (2 contigs, 15 singletons) were also identified, which encompassed transcriptional regulators, DNA or RNA binding proteins and translation elongation factors. All these proteins presumably reflect the metabolic effort invested by the venom gland for the transcription and translation of secreted products. The 2111 ESTs were used to produce *in silico *a database of venom gland open reading frames (vgORFs) which were matched to the peptide sequences obtained by nano-LC-MS/MS analysis. An overall of 1279 ESTs (26 contigs and 7 singletons, 60.6% of all ESTs) were then identified as coding for venom proteins of *C*. *inanitus*.

**Table 1:** Summary statistics of the analysis of the C. inanitus venom gland ESTs.

	No. of clusters	No. of ESTs	Percentage of total ESTs
**Total no. of contigs (clusters with > 1 member)**	**95**	**1718**	**81.38**
- coding for proteins identified by MS	26	1272	60.26
- coding for other proteins	69	446	21.13
			
**Total no. of singletons (clusters with 1 member)**	**393**	**393**	**18.62**
- coding for proteins identified by MS	7	7	0.33
- coding for other proteins	386	386	18.29
**TOTAL**	**488**	**2111**	**100**

### Identification of the venom proteins of *C. inanitus*

**Figure 2:**
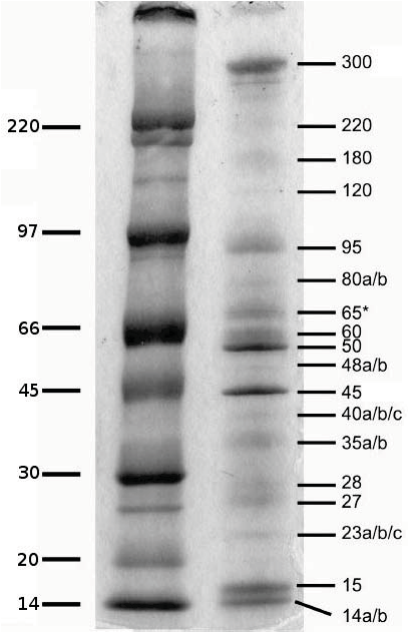
**SDS-PAGE of venom proteins from *C. inanitus***. Venom proteins collected from two venom reservoirs were separated on a 4-15% gradient SDS-PAGE gel and stained with Coomassie Brilliant Blue R-250. From such gels (run at varying conditions) bands were excised and submitted to nano-LC-MS/MS analysis. Numbers on the right indicate the approximate molecular mass of individual venom proteins for which amino acid sequences were obtained; only for 65, marked with an asterisks, no exploitable sequences were obtained. Molecular mass marker is shown on the left. This figure was modified from [38], with kind permission from Elsevier.

Venoms of parasitic wasps are reputed to have a low content in small proteins and peptides in comparison to venoms of social Hymenoptera [27,43]. Upon separation of *C. inanitus *venom proteins by SDS-PAGE, at least 25 proteins with apparent molecular masses ranging from 14 to 300 kDa had been observed while no bands were seen below 14 kDa. This was also consistent with data previously reported [38] from the analysis of SDS-PAGE and two-dimension electrophoresis gels stained with Silver or Coomassie blue staining methods. For the nano-LC-MS/MS analysis presented here, several gradient gels (4-15%) were run at various conditions to allow excision of all gel bands detectable upon Coomassie blue staining. Figure 2 shows that the analysed bands represent the majority of the venom proteins of *C. inanitus*. However, the presence in this venom of small amounts of additional proteins and peptides cannot be excluded. For 25 proteins, named Ci-14a to Ci-300, peptide sequences exactly matching sequences of the vgORF database were obtained upon nano-LC-MS/MS analysis (see additional file 1: Table of peptide identification). Furthermore, peptides belonging to four additional proteins were detected, namely Vem7, Vem11, Vem17 and Vem37 (Vem being an abbreviation for Venom Mix). These proteins were found in several gel bands, a situation usually found for very abundant proteins. There was no evident correspondence between the relative abundance of venom protein bands on the gel (Figure 2) and the abundance of the corresponding ESTs in the vgORFs database (Table 2). The detailed list of the identified venom proteins will be discussed in the following sections.

**Table 2:** List of venom proteins of C. inanitus identified by nano-LC-MS/MS analysis.

Name	Identification	EMBL Acc. No	Significant matches with Pfam entries	No. of corresponding ESTs	Seq. length (amino acids)
**Ci-23a**	Similar to venom protein from *C*. sp. near *curvimaculatus*	FN908672	-	534	114

**Ci-45**	Chitinase	FN908682	PF00704	64	387

**Ci-48b**	IDGF-like protein	FN908684	PF00704	118	440

**Ci-23c**	Mucin-like peritrophin	FN908674	PF01607 (×2)	22	177

**Ci-220**	Mucin-like peritrophin	FN908692	PF01607 (×2)	3	229

**Vem7**	Yellow-e3-like protein	FN908694	PF03022	15	432

**Ci-50**	Esterase/lipase-like	FN908685	PF00151	2	215^p^

Ci-300	Metalloprotease-like	FN908693	-	8	230^p^

**Ci-95**	ACE-like protein	FN908689	PF01401	19	145^p^

Ci-80a	C1A protease	FN908687	PF00112	1	165^p^

**Ci-40a**	Trypsin-like serine protease	FN908678	PF00089	2	144^p^

Ci-180	Lectin-like protein	FN908691	PF00059	2	218^p^

**Ci-14a**	CSP-like protein	FN908669	PF03392	1	104^p^

**Ci-23b**	Protein related to PBP/OBP	FN908673	PF01395	29	144

**Ci-48a**	Similar to lethal (1) G0193 isoforms	FN908683	-	5	383

Vem17	Similar to lethal (1) G0193 isoforms	FN908696	-	3	220^p^

**Ci-80b**	Similar to lethal (1) G0193 isoforms	FN908688	-	1	229^p^

**Ci-14b**	Unknown protein	FN908670	-	286	141

**Ci-15**	Unknown protein	FN908671	-	4	123

**Ci-27**	Unknown protein	FN908675	-	59	210

**Ci-28**	Unknown protein	FN908676	-	13	182

Ci-35a	Unknown protein	FN908677	-	5	188^p^

**Ci-35b**	Unknown protein	FN908678	-	5	266

Ci-40c	Unknown protein	FN908681	-	64	263^p^

Ci-60	Unknown protein	FN908686	-	2	205^p^

**Vem11**	Unknown protein	FN908695	-	3	217

**Vem37**	Unknown protein	FN908697	-	4	246

Ci-40b	Acidic ribosomal protein P0	FN908680	PF00466 PF00428	3	316^p^

Ci-120	alpha-N-acetyl glucosaminidase	FN908690	PF05089	2	165^p^

Protein names for which a signal peptide has been predicted are shown in bold; p, partial sequence.

### Ci-23a and Ci-45, two proteins with similarities with venom proteins from *C*. sp. near *curvimaculatus*

The Ci-23a venom protein was encoded by a contig corresponding to the highest number of ESTs in the library (534). It displays 50% of sequence identity (BlastP, E-value = 7e-21) with a 32.5 kDa protein referred as "venom protein from *C*. sp. near *curvimaculatus*" [GenBank:ACI70208.1], another chelonine wasp. This latter protein was historically the first to be isolated and sequenced from the venom of a parasitoid Hymenoptera [39]. Although it was found to be necessary for the survival of the parasitoid in the lepidopteran host, *Trichoplusia ni *[44], it is still not related to any other known protein and its function remains unknown to date. A potential cleavage site for both N-arginine dibasic convertase (pattern: .RK|RR[^KR]) and subtilisin-like proprotein convertase (pattern: [KR]R.) was detected at positions 51 to 53 (RRA) of the Ci-23a sequence. However, sequences coding for such convertases were not found in our vgORFs database. Remarkably, Ci-23a was devoid of the 12 tandem repeats of 14 residues that characterized the C-terminal part of the venom protein of *C*. sp. near *curvimaculatus *(see additional file 2: Amino acid sequence alignment of Ci-23a and the venom protein from *C. sp *near *curvimaculatus*) [39]. These repeat sequences form several α-helices with strong amphipathic structures supposed to run at the surface of the protein [39] and we found that they contain an unusualy high number of potential glycosaminoglycan attachment sites (14 serine motifs involved in the motif pattern: [ED]{0,3}.(S) [GA]). Thus Ci-23a, which is much shorter than its homologue, is potentially processed by non-venomous convertases and is likely to have a substrate or target site specificity different from that of the 32.5 kDa venom protein of *C*. sp. near *curvimaculatus*. Interestingly, the latter is a very abundant venom protein [44] while Ci-23a protein is of low abundance (Figure 2).

**Figure 3:**
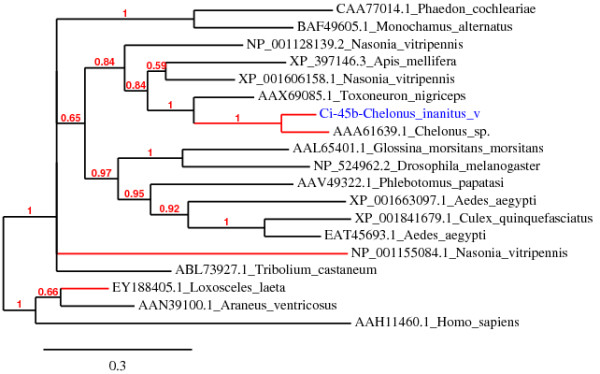
**Bayesian reconstruction of representative arthropod chitinase proteins**. The Ci-45 venom chitinase from *C. inanitus *characterized in this study is shown in blue and the branches of venom chitinases are shown in red.

The Ci-45 venom protein, which gives a strong band upon Coomassie staining (Figure 2), shows high similarity (BlastP, 79% identity, E-value = 2e-141) to a 52 kDa chitinase from venom of *C*. sp. near *curvimaculatus *[GenBank:AAA61639.1] [40]. Both proteins possess a predicted signal peptide and a glycosyl hydrolase family 18 domain (Pfam: PF00704) with four highly conserved regions present in all known insect chitinases [45-47] (see additional file 3: Amino acid sequence alignment of representative chitinases from different insect species). Members of glycosyl hydrolases 18 family show an eight-stranded α/β barrel catalytic core structure [48]. The 17 residues featuring this functional domain were all found in the Ci-45 sequence, notably those of the second conserved region implicated in catalysis, as shown by previous site-directed mutagenesis studies (consensus sequence: (F/L)DG(L/I)DLD(W/I)EYP)) [47,49]. Four cysteine residues involved in two disulfide bonds are conserved in the two Cheloninae enzymes and the secondary structures of both proteins were predicted to be highly similar in the placement of α-helix, β-strand and coil structure (data not shown). Ci-45 differs from its homologue by the absence of a C-terminal chitin-binding Peritrophin-A domain (CBM_14, Pfam: PF01607), but this domain does not appear to be essential for the chitinolytic activity of chitinases in Arthropods [46,50-52]. The Ci-45 contig is thus most likely coding for an active venom chitinase and might be responsible for the chitinase activity previously detected in the venom of *C. inanitus *[38]. According to the classification proposed by Zhu *et al*. [47], it belongs to a group of conserved insect chitinases containing a single catalytic domain (group IV). Our phylogenetic analysis (Figure 3) grouped the chitinases from venom glands of *C. inanitus *and *C*. sp. near *curvimaculatus *in a monophyletic clade with that from teratocytes of *T. nigriceps *[GenBank:AAX69085.1]. Teratocytes are parasitoid wasp secretory cells that circulate into the parasitized host haemolymph, and represent a different way for the wasp to deliver virulence factors into the host. The teratocyte released chitinase from *T. nigriceps *is hypothesized to contribute to the avoidance of microbial contamination of the host's haemocoel or to facilitate the emergence of parasitoid larvae through the host's cuticle [53]. Other examples of chitinases produced by venom glands were previously reported from spiders [54,55] and from the parasitoid wasps *M. hyperodae *[33] and *N. vitripennis *[28]. More recently, a chitinase produced by the posterior salivary glands of the cephalopod *Octopus kaurna *has also been described [56]. Venom and salivary glands chitinases described are thus restricted to invertebrate species. Interestingly, our phylogenetic analysis shows that venom chitinases belong to distinct clades comprising also non venomous enzymes. This suggests chitinases have been selected for production in invertebrate venom glands through multiple independent recruitment events.

### Ci-48b, an IDGF-like protein

Like Ci-45, Ci-48b possesses a glycosyl hydrolase family 18 domain (see additional file 4: Amino acid sequence alignment of Imaginal disc Growth Factors (IDGFs)-like proteins from different insect species). However, in the second conserved region, a glutamine residue (Q157) replaces the glutamic acid residue that plays a key role in active insect and bacterial chitinases, in being the putative proton donor during the catalytic mechanism [49,57,58]. This feature is shared by members of the imaginal disc growth factors (IDGFs) family which have presumably evolved from chitinases to gain new functions [59]. Interestingly, Ci-48b shows high sequence similarity to IGDFs from several insect species, namely a venom gland protein of honey bee workers [NCBI Reference Sequence: XP_396769.2] and a hemocyte aggregation inhibitor protein from the lepidopteran species *Manduca sexta *[GenBank:ACW82749.1] (Blast P 38% identity; E-value = 2e-76) [60]. Thus Ci-48b may contribute to the survival and development of *C. inanitus *eggs once oviposited into their host either by acting as a growth factor or alternatively, by modulating the cellular immune response of young *S. littoralis *host larvae.

### Ci-23c and Ci-220, two putative mucin-like peritrophins

The sequence of the Ci-23c venom protein is 177 amino acid long, giving a theoretical molecular mass of 19.6 kDa (see additional file 5: Amino acid sequence alignment of Ci-23c, Ci-220 and AD-873). It possesses two Chitin-binding peritrophin domains 14 (CBM_14, positions 26-78 and 92-144) and a predicted glycosaminoglycan attachment site (positions 155-158). Another venom protein, Ci-220 (229 amino acids, theoretical molecular mass 26.3 kDa), also contains two CBM_14 domains (positions 29-84 and 82-135) and a predicted glycosaminoglycan attachment site (positions 138-141). The two proteins share an overall 32% identity (BlastP, E-value = 1e-16) and show sequence similarities to a wide variety of CBM_14 domains containing proteins, including a venom component from *N. vitripennis *[28]. Moreover they share the domain organization of the mucine-like peritrophin AD-873 identified from the salivary gland transcriptome of the mosquito *Anopheles darlingi *[GenBank:ACI30179.1]. Interestingly this peritrophin is speculated to contribute to the maintenance of the structure of the mouthparts and/or salivary canal of *A. darlingi *[61]. In *C*. *inanitus*, oviposition is accompanied by intense contractions of the abdomen, which are necessary to push the venom from the reservoir into the oviduct, since the venom reservoir is located at the distal end of one of the gland filaments and has a very thin wall without a muscle layer [38]. It is thus possible that the venom proteins Ci-23c and Ci-220, as the mosquito peritrophin, contribute to keeping the reservoir in shape.

### Vem7, an ancient yellow-e3-like venom protein

**Figure 4:**
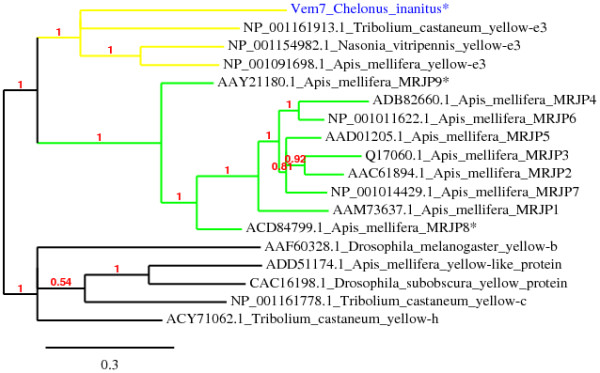
**Bayesian reconstruction of representative yellow-e3 and MRJPs proteins**. The Vem7 protein from *C. inanitus *characterized in this study is shown in blue. Yellow proteins clades are shown in yellow and MRJPs clades are shown in green. Asterisk indicates protein expressed by venom glands.

Vem7 shares high sequence similarity to the yellow-e3 protein [GenBank:ABB82366.1] from *A*. *mellifera *(BlastP, 42% identity, E-value = 2e-81). The *yellow-e3 *gene is highly expressed in the head and hypopharyngeal gland of honey bee workers and is considered as the progenitor of all genes of major royal jelly proteins (MRJPs) of *A. mellifera*. Located in the same genomic region and sharing a similar exon/intron organization, MRJPs would have been generated *via *recent duplications [62,63]. Recently, two members of the MRJPs family, MRJP8 [GenBank:ACD84799.1] and MRJP9 [GenBank:ACD84800.1], were identified in the venom gland proteome of *A. mellifera *[5]. It is noteworthy that phylogenetic analyses put them at the basis of the MRJPs tree, meaning that they are the most ancient members of the MRJPs family and suggesting a venomous "pre-royal jelly" function for the MRJPs progenitor originating from yellow-e3 [5,63]. Our phylogenetic analysis shows that Vem7 forms a monophyletic group with yellow-e-3 protein (Figure 4) and thus represents the first example of a venomous protein in this clade. This supports the hypothesis that a yellow-e-3 gene progenitor of MRJPs may have encoded a venomous protein like the *Vem7 *gene.

### Putative enzymes

Ci-50 is a venom protein belonging to the lipase family (Pfam: PF00151). One carboxyl-esterase and two other lipases were previously identified in the venom proteome of *N. vitripennis *[28] and a lipase activity has been found in the venom of *P. hypochondriaca *[64]. In *N. vitripennis*, venom lipases might participate in the alteration of host's lipid metabolism to the benefit of the developing parasitoid eggs [65] and a similar function is conceivable for *C. inanitus*.

The partial sequence of the Ci-300 venom protein shares 39% sequence similarity (BlastP, E-value = 2e-05) with a Zinc-dependent metalloprotease identified in the venom of *P. hypochondriaca *[GenBank:CAD21587.1]. However, Ci-300 lacks the functional characteristic Zn^2+^-binding motif of HExxHxxGxxH and a distal located methionine [66] found in venom metalloproteinases from the parasitoid species *P. hypochondriaca *[67], *M. aethiopoides *[33], *Eulophus pennicornis *[68] and *N*. *vitripennis *[28] (see additional file 6: Partial amino acid sequence alignment of Ci-300 with insect metalloproteases). The sequence of Ci-300 could have thus considerably diverged from an ancestral Zinc-dependent metalloprotease-like protein to acquire an original function in the venom of *C*. *inanitus*.

The Ci-95 venom protein shows significant sequence similarity to various angiotensin converting enzymes (ACEs, Pfam: PF01401) and notably to an ACE-like protein from *A. mellifera *[NCBI Reference Sequence:XP_393561.2] (BlastP, 46% identity, E-value = 2e-29). In addition, upon Western analysis with an antibody made against recombinant Drosophila ACE (kindly provided by Dr. Elwyn Isaac, University of Leeds, UK) a clear band was seen (data not shown). ACE is a dipeptidyl carboxydipeptidase with a broad *in vitro *substrate specificity that is best known, in mammals, for its role in converting inactive angiotensin I to the vasoconstrictor, angiotensin II, and the inactivation of bradykinin [69]. In insects, ACE-like proteins appear to have a wide tissue distribution, from embryos to adult stages [70,71] and some have been implicated in the metabolic inactivation of neuropeptides in the central nervous system [72]. Dani *et al*. [73] have detected an ACE-like enzymatic activity in the venom of *P. hypochondriaca *and have speculated that venomous ACE could be involved in the processing of peptide precursors in the venom reservoir. More recently, another ACE-like enzyme has also been identified in the venom of *N. vitripennis *[28].

Ci-80a, a venom protein encoded by a single transcript identified in our VgORFs database, belongs to the peptidase family C1, sub-family C1A (papain family, clan CA, Pfam: PF00112). The partial sequence of Ci-80a contains two out of the four conserved residues of the active site of C1A proteases and all the residues involved in the S2 subsite, which is involved in specificity for the dominant substrate of papain-like cysteine proteases. Interestingly, viral cystatins encoded by the genome of the bracovirus CcBV, associated with the parasitic wasp *Cotesia congregata*, were shown to target some C1A proteases of the host *M. sexta *[74]. Cathepsins and their inhibitors may play an important role, yet undetermined, in the context of host-parasitoid physiological relationships.

The Ci-40a venom protein contains a partial trypsin-like serine protease domain (Pfam: PF00089) but displays low sequence similarities with other known proteases (see additional file 7: Amino acid sequence alignment of Ci-40a with serine protease homologs (SPHs)). At least one of the three residues involved in the catalytic triad for serine protease (aspartate residue in position 111) is present in the partial sequence of Ci-40a. Several serine protease homologs were already reported from parasitoid venoms. In the endoparasitoid *C. rubecula*, the venom protein Vn50 [GenBank:AAP49428.1] has been found to be a mutated serine protease acting as an inhibitor of the defensive reaction of melanization of host hemolymph [21]. In addition, members of the serine protease protein family were recently identified in the venom proteomes of *N. vitripennis *[28] and *P. puparum *[29].

The Ci-120 protein shares significant sequence similarity with insect alpha-N-acetyl glucosaminidases (Pfam: PF05089, EC: 3.2.1.50) and might play a role in proteoglycan metabolism.

### Lectin-like venom protein

A C-type lectin domain (Pfam: PF00059) was found in the sequence of the Ci-180 venom protein. The domain extends from positions 76 to 199 of the partial sequence. An immunosuppressive function has been proposed for a lectin with a similar C-type lectin domain, encoded by the genome of the bracovirus CpBV, associated with the parasitic wasp *Cotesia plutellae *[75]. Since parasitoid venoms and PDVs are used by the wasps to manipulate parasitized host physiology, it might not be surprising that common molecules have been selected for delivery into the host *via *different pathways.

### CSP-like and OBP-like proteins

The Ci-14a venom protein belongs to the A10/OS-D insect pheromone-binding protein family (Pfam: PF03392). A high sequence similarity was observed between Ci-14a and Csp3 from *A*. *mellifera *(BlastP, 56% identity, E-value = 5e-27), another member of the A10/OS-D protein family. Csp3 [GenBank:ABH88171.1] is a chemosensory protein (CSP) ubiquitously expressed in adults and pre-imaginal stages of the honeybee in which it may play a role in cuticle maturation [76].

Ci-23b is a venom protein containing a pheromone binding protein/general-odorant binding protein (PBP/GOBP) domain (Pfam: PF01395). Homology searches revealed that Ci-23b has highly diverged from known PBPs and GOBPs. In particular, it only shows 4 out of the 6 cystein residues strictly conserved between PBPs and GOBPs [77,78]. Other OBP-like venom proteins, unrelated to Ci-23b, have previously been identified in the venom proteomes of *A. mellifera *workers and *N*. *vitripennis *females (NCBI Reference Sequences: NP_001035313.1 and NP_001155150.1, respectively) [5,28]. Beside the involvement of PBPs and OBPs in chemical communication, it is possible that in Hymenoptera, some OBP-like proteins fulfil other roles in relation with the venomous functions.

### Venom proteins similar to lethal (1) G0193 isoforms

Ci-48a, Vem17 and Ci-80b venom proteins share sequence similarities with members of a group of proteins similar to protein isoforms A [GenBank:AAF46301.1] and B [GenBank:AAS65275.1] encoded by the *lethal (1) G0193 *gene from *D. melanogaster*. This group of cystein-rich proteins notably includes several venom and salivary gland proteins of unknown functions reported from various insect species (see additional file 8: Sequence similarities between Ci-48a, Vem17 and Ci-80b and proteins similar to isoforms of lethal (1) G0193). Ci-80b possesses the least conserved amino acid sequence towards lethal (1) G0193 isoforms suggesting it might have diverged as a virulence factor involved in host-parasite interactions, which are often characterized by a high level of divergence.

### New lineage-specific proteins

In addition to Ci-23a and Ci-45 which are conserved in the venom of two *Chelonus *species, ten *C*. *inanitus *venom proteins did not show any significant sequence similarity to known proteins (Ci-14b, Ci-15, Ci-27, Ci-28, Ci-35a, Ci-35b, Ci-40c, Ci-60, Vem11 and Vem 37). This is reminiscent of observations on twenty three venom proteins in *N. vitripennis *which also have no similarity to known proteins and appear to be lineage- and/or species specific [28]. Given that Chelonines are egg-larval parasitoids it is possible that new *C. inanitus *proteins have evolved to cope with this particular parasitic context. Although they do not contain conserved domains, they may play an important role during host-parasite interaction and notably Ci-14b, the second most abundant sequence of the transcriptome (286 ESTs).

### Additional putative venom proteins identified from the vgORFs database

**Figure 5:**
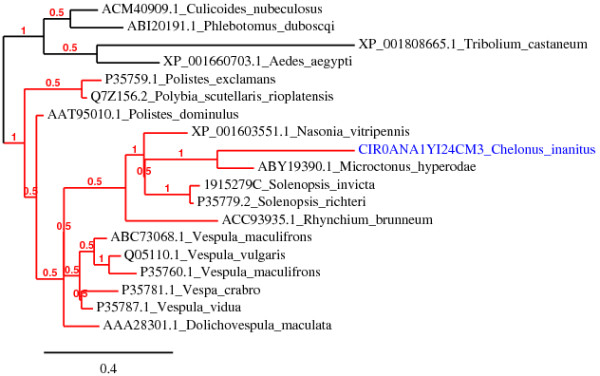
**Bayesian reconstruction of representative Allergen 5 proteins**. The EST from *C*. *inanitus *encoding an allergen 5-like protein and characterized in this study is shown in blue. Venom allergen 5 proteins clades are shown in red.

In addition to proteins identified by mass spectrometry, several ESTs encoding putative venom proteins were identified in our vgORFs database (Table 3). Five ESTs encoded partial sequences of at least three different hyaluronidase-like proteins that show significant sequence similarities to venom hyaluronidases described from several hymenopteran species [79]. One EST encoded a venom allergen 5-like protein (Figure 5). Venom allergen 5 proteins (also called antigens 5) are commonly found in venoms of social Hymenoptera of the superfamilly Vespoidea [80]. They belong to a wider group of proteins expressed by salivary and venom glands of distant animal species, recently gathered under the proposed term of CAP proteins for "Cystein-rich secretory proteins, Antigen 5 and Pathogenesis-related proteins" [56]. CAP domain proteins are the dominant allergy-inducing toxins in hymenopteran venoms [81], and a related CAP protein has previously been identified from the venom of *M. hyperodae *[33].

**Table 3:** Additional putative venom proteins identified by similarity searches (BLASTP).

Sequence name	**EMBL Acc. no**.	Best-matched Protein (Acc. no.)	Species	Id. (%)	E-value
A6YN21CM1	FN985040	Hyaluronidase [GenBank:ACD61711.1]	*Orancistrocerus drewseni*	37	2e-16

A4YE08CM1	FN985041	Hyaluronidase [GenBank:AAA27730.1]	*Apis mellifera*	46	7e-07

A3YM10CM1	FN985042	Hyaluronidase 2 [GenBank:AAK51798.2]	*Apis cerana cerana*	45	8e-36

A2YM17CM2	FN985043	Hyaluronidase [GenBank:BAF93867.1]	*Anoplius samariensis*	37	6e-13

A1YB14CM3	FN985044	Hyaluronidase [GenBank:AAA27730.1]	*Apis mellifera*	42	7e-43

A1YI24CM3	FN985045	Allergen 5 [GenBank:AAA28303.1]	*Dolichovespula arenaria*	48	7e-36

Amino acid sequences encoded by the mentioned ESTs were compared to protein sequences deposited in NCBI nr database using BLASTP algorithm. Id.: identity between the amino acid sequence deduced from a given *C. inanitus *transcript and the corresponding best-matched protein.

## Conclusions

In this paper we report the identification of the majority of venom proteins of the egg-larval endoparasitoid wasp *C. inanitus*. We combined technical approaches which we had successfully used to elucidate the origin of bracoviruses [82]. They are powerful tools to study evolutionary and functional aspects of parasitoid-associated factors. The most highly redundant set of sequences encoded a protein (Ci-23a) that shared sequence similarity with a venom protein previously identified in the related species *C*. sp. near *curvimaculatus *[39]. These venom proteins are thus likely to play a key role, as yet undetermined, in the life cycle of Cheloninae egg-larval endoparasitoids. In addition, we have identified 453 unigenes that, for the most part, are likely to code for nonsecretory products of the venom glands.

A striking feature of *C. inanitus *venom was the redundancy of components able to interact with chitin. These components might be important when intermediate or late stages of eggs are parasitized. In this case, the parasitoid larva has to bore itself into the host embryo which is surrounded by an embryonic cuticle [83] and chitinases might help to facilitate this process.

A number of *C. inanitus *venom proteins and enzymes shared similarities to venom gland products from other species that also belong to the Ichneumonoidea superfamily (*M. hyperodae*, *M*. *aethiopoides, P. hypochondriaca*). Sequence similarities were also found with venom proteins from more distant apocritan species representative from Chalcidoidea (*N. vitripennis*), Vespoidea (*O*. *drewseni, Anoplius samariensis, Dolichovespula arenaria*) and Apoidea (*A. mellifera, Apis cerana cerana*) superfamilies (see additional file 9: Phylogeny of the major superfamilies of Hymenoptera). The presence of related venom proteins in species that do not share the same ecological constraints and lifestyles can partly be explained by independent recruitment of these proteins during species evolution [56]. Our phylogenetic analyses suggest this is the case for the venom chitinases of the Cheloninae species and the venom chitinase 5 of *N. vitripennis *that were acquired independently. However, given that all modern Apocrita share a common ancestral parasitic origin [84], it is also expected that some lineages have conserved ancestral venom genes. Our finding of mRNA coding for a member of the allergen 5 proteins family in the venom glands of *C. inanitus *appears to be an example of such conservation. The sequence of the deduced protein is placed, with the allergen 5 from the venom of *M. hyperodae *and *N. vitripennis *in a monophyletic clade with respect to the phylogenetic tree of allergen 5 proteins found in vespid and ant venoms (Figure 5). This result suggests that the ancestral gene was expressed by the venom glands of the common ancestor of Ichneumonoidea and Aculeata, 155 to 185 million years ago [84] and could have been lost in Apoidea. Allergen 5 proteins would thus be representative of one of the most ancient group of insect venom proteins.

Another interesting point is the discovery of a yellow-e3-like protein, Vem7, in the venom of *C*. *inanitus *which give more indications on the evolutionary history of the yellow-e3 gene family among Hymenoptera. Interestingly the recent genome sequencing of *N. vitripennis *has revealed the largest number of yellow/MRJP genes so far found in any insect, including an independent amplification of MRJP-like proteins [85]. It would be worthy to determine if some of these genes have a venomous function in *Nasonia *species.

On a more general standpoint, once increasing number of comprehensive analyses will become available, our work on the venom composition of *C. inanitus *will contribute to retracing the evolution of venomous functions within Hymenoptera by comparison of the venomous arsenal of different species.

## Methods

### Insects

*C. inanitus *(Hymenoptera: Braconidae), a solitary egg-larval parasitoid, was reared on one of its natural hosts *S. littoralis*. Adult *S. littoralis *were kindly provided by Syngenta AG, Stein, Switzerland. They were raised at 27°C at a LD photoperiod of 14 h:10 h and fed with an artificial diet. Diet was prepared from dry powder (Beet Army Worm Diet, Bio Serv, Frenchtown, New Jersey, USA).

### Collection of venom glands and RNA isolation

Female wasps were anaesthetized on ice for several minutes and then shortly rinsed in 70% ethanol. The abdominal organs were gently pulled out with forceps and the reproductive apparatus was placed in 50 μl sterile Insect Ringer. Then, the venom gland filaments were dissected, washed in sterile Ringer, put into an Eppendorf tube containing 200 μl of RNAlater™(Qiagen) and stored at - 80°C until RNA isolation. The morphology of the *C. inanitus *venom gland was previously described by Kaeslin *et al*. [38]. For RNA isolation, venom gland filaments from 60 female wasps were used; no homogenization was necessary and 0.45 ml Lysis buffer RLT (Qiagen) including 143 mM β-mercaptoethanol was added followed by a proteinase K digestion as described by Johner and Lanzrein [86]. The RNA isolation (RNeasy Mini Kit, Qiagen) including an on column DNase digestion (RNase-free DNase I) was performed according to the manufacturer's protocol. Total RNA was eluted from the column with 100 μl RNase-free water. A second digestion with DNase I was carried out and the RNA was then extracted with acidic phenol; the RNA was precipitated as described in [87]. The yield was 9 μg of total RNA.

### cDNA library construction

A cDNA library was constructed with the Creator SMART cDNA Library Construction Kit (BD Biosciences, Ozyme, France) following the supplier's instructions. The first strand cDNA was synthesized from 481 ng of total RNA extracted from *C. inanitus *venom glands. The cDNA were ligated into pDNR-LIB (BD Biosciences Clontech) and ligation products were transformed into ElectroMax DH10B-T1 Phage Resistant *Escherichia coli *Competent Cells (Invitrogen, Fisher Scientific, France).

### Sequencing and ESTs quality control

To obtain an unbiased overview of the venom gland transcriptome, colonies were amplified with the ϕ29 DNA polymerase by rolling circle amplification. Sequencing was done on a ABI sequencer using the standard M13 forward primer and BigDye terminator cycle sequencing kit (Applied Biosystems, Foster City, CA, USA). Initially 384 clones from the library were analyzed for the presence of venom proteins-related sequences. These were detected and a sequencing of a total of 2500 clones was performed. The EST sequences obtained were analysed for quality control; base calling step was performed with the Phred program f(S9). Low quality bases (phred score < 20) were masked and sequences with more than 30% n-content, or shorter than 60 bp (after vector/adaptator sequences removing) were removed.

### ESTs clustering and assembly

After discarding the poor-quality sequences, 2111 high-quality ESTs were subjected to clustering using the TIGR software TGI Clustering tool (TGICL) [88]. The clustering was performed by a modified version of NCBI's megablast. EST sequences were assigned to clusters based on identity: the clustering parameters were 98% minimum percent identity for overlaps, for a minimum overlap length of 40 nt and a maximum length of unmatched overhangs of 20 nt.

Sequences from each cluster were assembled into consensus sequences called "contigs" using the CAP3 assembly program [89] available in TGICL. Sequences from a cluster containing only one sequence were called "singletons".

### ESTs annotation

To identify similarities with known proteins, the sequences of contigs and singletons were searched using the BLASTX algorithm against a local non-redundant protein database (NR, NCBI) with a cut-off E-value of 1e-5. To define the function of the contigs and singletons, we used the Gene Ontology (GO) controlled vocabulary [90] and more particularly GOSlim, a subset of GO terms, which provides a higher level of annotations and allows a more global view of the dataset. To this end, an automated GO-annotation of the sequences of contigs and singletons that showed a significant similarity with a Uniprot entry was achieved using the Blast2go software [91], with a stringency cut-off of 1e-6.

### Collection of venom, SDS-polyacrylamide gel electrophoresis and protein identification

Wasps were anaesthetized on ice and their venom apparatus (venom gland filaments and reservoir) was dissected. For each protein separation the venom of four wasps was collected. The venom was collected by piercing the reservoirs with a glass capillary. The sucked up venom was collected in 10 μl sterile H_2_O and mixed with 10 μl sample buffer (0.125 M Tris-HCl pH 6.5, 4% (w/v) SDS, 10% (v/v) glycerol, 0.01% bromophenol blue, 5% (v/v) β-mercaptoethanol) and heated at 90°C for 6 minutes. For separation of proteins, precast linear gradient READY GEL (BIO RAD) 4-15%Tris-HCl gels with 10 wells were used. Two equivalents of venom (i.e. the amount of venom collected from two reservoirs) were loaded per lane. Electrophoresis was in 0.02 M Tris-HCl pH 8.8, 0.19 M glycine, and 0.1% (w/v) SDS. A constant voltage of 150 V was applied. As marker the High-Range Rainbow™Molecular Weight Marker (GE Healthcare) was used. Staining was done with SERVA blue R (Coomassie) Brilliant Blue R-250 according to the manufacturer's protocol. For protein identification, gel slices were cut out, transferred into Eppendorf tubes and covered with 20 μl ethanol (20%). Treatment of gel slices and nano-LC-MS/MS analysis was as described in [82]. CID spectra were extracted into data files by Bioworks (Rev.3.3.1, Thermo Scientific) without any filters applied. Combined dta files were automatically matched to our personal database of vgORFs by Phenyx software, Version 2.5 (Genebio SA, Geneva, Switzerland). N-terminal sequencing was done by Edman degradation.

### Sequence analysis

Each cluster of nucleotide sequences was annotated by being searched against GenBank NCBI database [92] with BLAST algorithms. Since amino acid sequences are more useful to detect homology over long periods, the assembled sequences were translated on-line into the correct open reading frames (ORFs) using ORFINDER tool from NCBI [93] and compared to the sequences in the NCBI nr and Swissprot protein databases. Sequences that did not match were further compared against the Genbank nucleotide databases (Blastn).

The signalP algorithm [94] was accessed online to predict the presence of signal peptides. The deduced amino acid sequences of all the proteins identified by nano-LC-MS/MS analysis were annotated by searching against Pfam protein families database [95]. Modification, cleavage and functional sites were predicted by the ELM server [96].

For amino acid sequence alignments, sequences were retrieved from NCBI nr database, aligned with sequences from *C. inanitus *with the program MAFFT version 6.0 [97] and edited with the program Jalview [98]. Alignment refinement was done with Gblocks software (version 0.91b) [99]. Phylogenetic relationships were estimated by Bayesian MCMC analyses using the program MrBayes 3.1.2 [100] available online [101,102]. For each set of aligned sequences, we implemented a mixed model of amino acid substitution, with gamma-correction for heterogeneity rate among residues and correction for invariable residues. Model of protein evolution was selected using ProtTest 2.4 software [103].

## Authors' contributions

BV and SM carried out the cDNA library construction, sequence alignments and annotations, performed the phylogenetic analyses and drafted the manuscript. TR isolated the venom gland RNA and participated in the preparation of venom proteins for sequencing. MK participated in the preparation of venom proteins and their sequence analysis. MH carried out the LC-MS/MS analyses and CID spectral interpretation. JS carried out the N-terminal sequencing. JP coordinated ESTs sequencing and quality controls. FC performed ESTs clustering and assembling. BL coordinated the proteomic study and the extraction of RNA from *C. inanitus *venom gland. JMD and MP participated in the design and coordination of the transcriptomic study. All authors read and approved the final manuscript.

## Supplementary Material

Additional file 1 **Table of peptide identification**. Peptidic sequences obtained from the nano-LC-MS/MS analysis of venom proteins from *C. inanitus *are shown in blue into the corresponding amino acid sequences encoded by ORFs obtained from the transcriptome analysis of *C. inanitus *venom glands. Click here for file

Additional file 2 **Amino acid sequence alignment of Ci-23a and the venom protein from *C. sp *near *curvimaculatus***. The amino acid sequence of the venom protein from *C. sp *near *curvimaculatus *was retrieved from GenBank [GenBank:ACI70208.1]. The position of a potential cleavage site for both N-arginine dibasic convertase and subtilisin-like proprotein convertase is boxed in black in the Ci-23a sequence. Red and green arrows indicate the beginning of the predicted signal peptide and mature protein sequences of Ci-23a, respectively. Serine residues that potentially serve as glycosaminoglycan attachment sites are indicated by blue triangles under the sequence of the venom protein from *C. sp *near *curvimaculatus*. Sequence printed in bold was also obtained by N-terminal sequencing of the Ci-23a protein. Click here for file

Additional file 3 **Amino acid sequence alignment of representative chitinases from different insect species**. The sequence of the Ci-45 venom chitinase from *C. inanitus *was aligned with sequences of chitinases from the following species: the parasitic wasps *C*. sp. near *curvimaculatus *[GenBank:AAA61639.1], *T. nigriceps *[GenBank:AAX69085.1] and *N. vitripennis *[GenBank:NP_001128139.2] and the beetle *Monochamus alternatus *[GenBank:BAF49605.1]. The four conserved regions are boxed. Black triangles indicate catalytic residues. Locations of the glycosyl hydrolase family 18 and chitin-binding Peritrophin-A (CBM_14) domains are indicated by blue and green lines, respectively. Red and green arrows indicate the beginning of the predicted signal peptide and mature protein sequences of Ci-45, respectively. Sequence printed in bold was also obtained by N-terminal sequencing of the Ci-45 protein. Click here for file

Additional file 4 **Amino acid sequence alignment of Imaginal disc Growth Factors (IDGFs)-like proteins from different insect species**. The sequence of the Ci-48b from *C. inanitus *was aligned with sequences from the following species: *N. vitripennis *[GenBank:XP_001599305.1], *A. mellifera *[GenBank:XP_396769.2] and *Manduca sexta *[GenBank:ACW82749.1]. The conserved region II is boxed. Triangle indicates a glutamine residue replacing, in these proteins, a glutamic acid residue of functional importance. Location of the glycosyl hydrolase family 18 domain is indicated by a blue line. Red and green arrows indicate the beginning of the predicted signal peptide and mature protein sequences of Ci-48b, respectively. Sequence printed in bold was also obtained by N-terminal sequencing of the Ci-48b protein. Click here for file

Additional file 5 **Amino acid sequence alignment of Ci-23c, Ci-220 and AD-873**. The sequences of the Ci-23c and Ci-220 proteins from *C. inanitus *were aligned with the sequence of the AD-873 protein from *Anopheles darlingi *[GenBank:ACI30179.1]. Location of the chitin-binding Peritrophin-A (CBM_14) domains of Ci-23c are indicated by green lines under the alignment. Serine residues that potentially serve as glycosaminoglycan attachment sites are indicated by blue triangles. Red and green arrows indicate the beginning of the predicted signal peptide and mature protein sequences of Ci-23c, respectively. Click here for file

Additional file 6 **Partial amino acid sequence alignment of Ci-300 with insect metalloproteases**. The sequence of Ci-300 was aligned with sequences of metalloproteases from the following species: *N. vitripennis *[NCBI Reference Sequence:XP_001604431.1] and [NCBI Reference Sequence: NP_001155006.1], *P. hypochondriaca *[GenBank:CAD21587.1] and *E. pennicornis *[GenBank:ACF60597.1]. The Zn^2+^-binding motif of HExxHxxGxxH featuring known metalloproteases' partial alignment is boxed in black. Click here for file

Additional file 7 **Amino acid sequence alignment of Ci-40a with serine protease homologs (SPHs)**. The partial sequence of Ci-40a was aligned with sequences of SPHs from the following species: *A. mellifera *[NCBI Reference Sequence:XP_623150.2], *C. rubecula *[GenBank:AAP49428.1], *N. vitripennis *[NCBI Reference Sequence:NP_001166254.1] and [NCBI Reference Sequence:NP_001155014.1] and *A. aegypti *[NCBI Reference Sequence:XP_001661226.1]. The location of the trypsin-like serine protease domain of Ci40a is indicated by a blue line under the alignment. Click here for file

Additional file 8 **Sequence similarities between Ci-48a, Vem17 and Ci-80b and proteins similar to isoforms of lethal (1) G0193**. Sequences similarities were determined by comparing the amino acid sequences of Ci-48a, Vem17 and Ci-80 to protein sequences deposited in NCBI nr database using BLASTP algorithm. The percentage of identity and E-value are given for each comparison. E: embryo; n.d.: not determined; n.s.: no significant similarity found (E-value> 1e-04); SG: salivary gland; VG: venom gland. Click here for file

Additional file 9 **Phylogeny of the major superfamilies of Hymenoptera**. Family and species names discussed in the present paper are indicated on the right side of the figure. The phylogeny of Hymenoptera shown on the left side of the figure is adapted from [9]. Click here for file
