# Supplementary figures and images for: The venom composition of the parasitic wasp Chelonus inanitus resolved by combined expressed sequence tags analysis and proteomic approach
Source: BMC Genomics. 2010 Dec 7;11:693. doi: 10.1186/1471-2164-11-693 (PMC3091792; doi:10.1186/1471-2164-11-693)

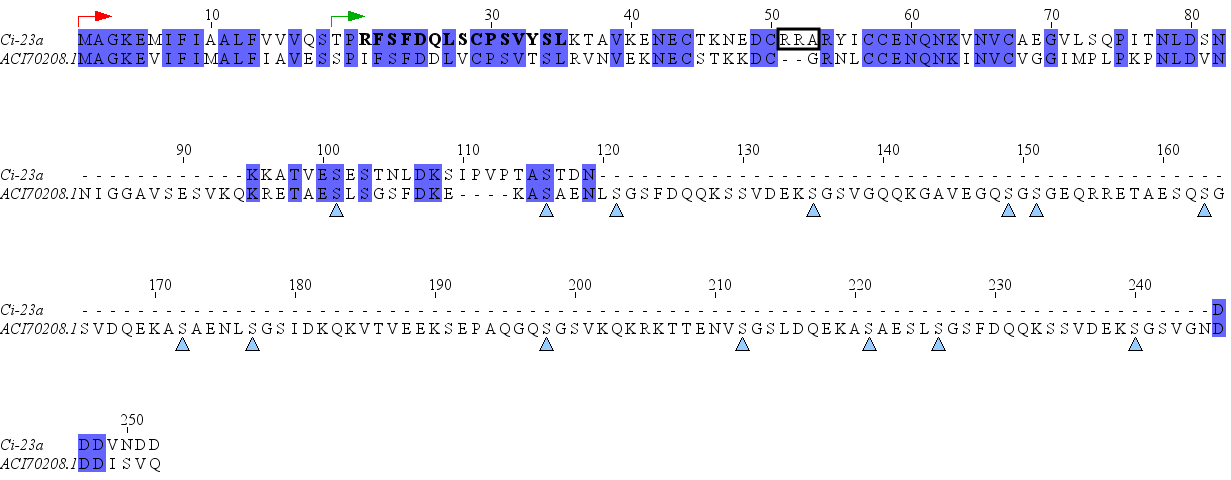

Supplement: Additional file 2 — Amino acid sequence alignment of Ci-23a and the venom protein from C. sp near curvimaculatus. The amino acid sequence of the venom protein from C. sp near curvimaculatus was retrieved from GenBank [GenBank:ACI70208.1]. The position of a potential cleavage site for both N-arginine dibasic convertase and subtilisin-like proprotein convertase is boxed in black in the Ci-23a sequence. Red and green arrows indicate the beginning of the predicted signal peptide and mature protein sequences of Ci-23a, respectively. Serine residues that potentially serve as glycosaminoglycan attachment sites are indicated by blue triangles under the sequence of the venom protein from C. sp near curvimaculatus. Sequence printed in bold was also obtained by N-terminal sequencing of the Ci-23a protein. [file 1471-2164-11-693-S2.PNG]

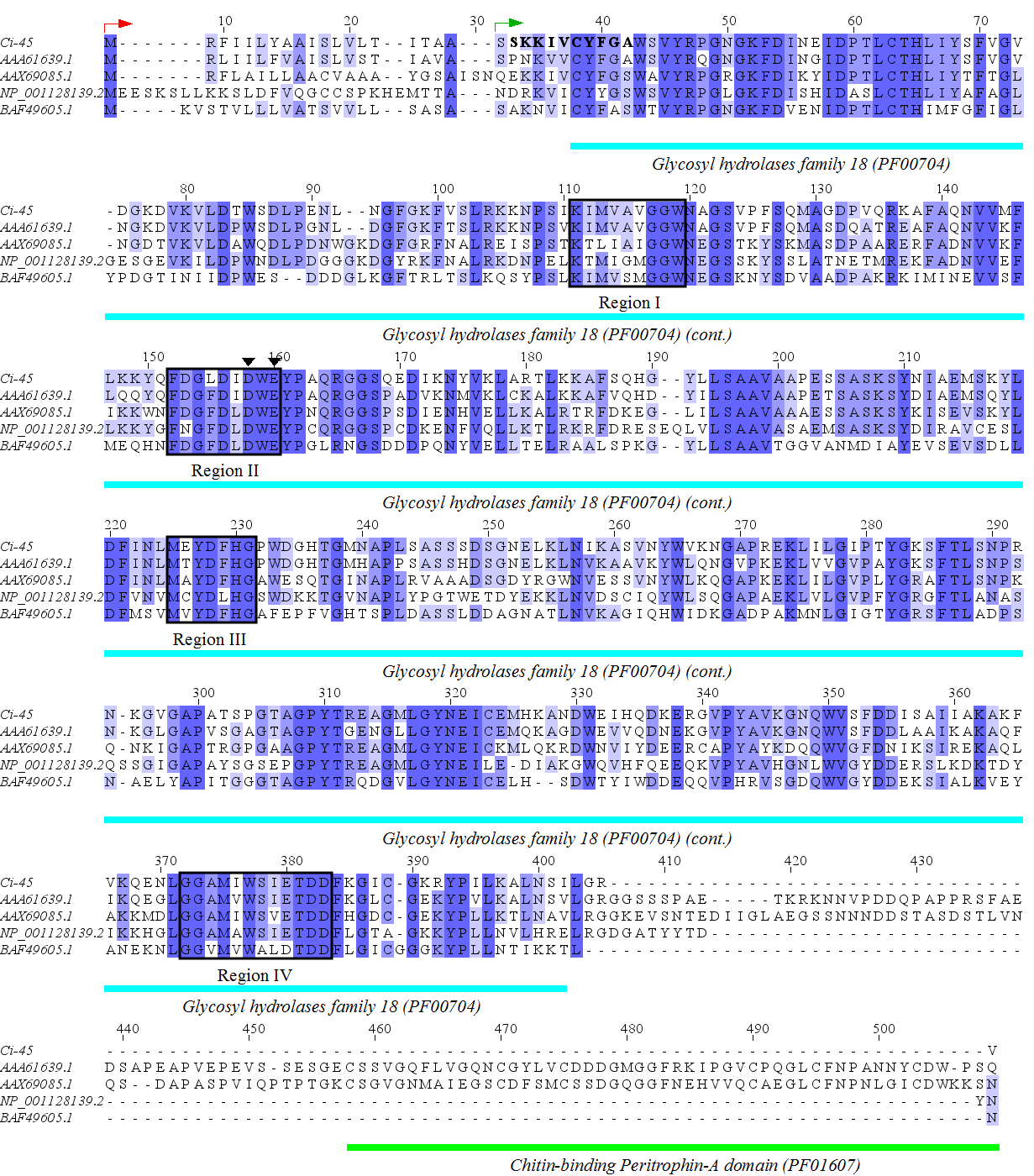

Supplement: Additional file 3 — Amino acid sequence alignment of representative chitinases from different insect species. The sequence of the Ci-45 venom chitinase from C. inanitus was aligned with sequences of chitinases from the following species: the parasitic wasps C. sp. near curvimaculatus [GenBank:AAA61639.1], T. nigriceps [GenBank:AAX69085.1] and N. vitripennis [GenBank:NP_001128139.2] and the beetle Monochamus alternatus [GenBank:BAF49605.1]. The four conserved regions are boxed. Black triangles indicate catalytic residues. Locations of the glycosyl hydrolase family 18 and chitin-binding Peritrophin-A (CBM_14) domains are indicated by blue and green lines, respectively. Red and green arrows indicate the beginning of the predicted signal peptide and mature protein sequences of Ci-45, respectively. Sequence printed in bold was also obtained by N-terminal sequencing of the Ci-45 protein. [file 1471-2164-11-693-S3.PNG]

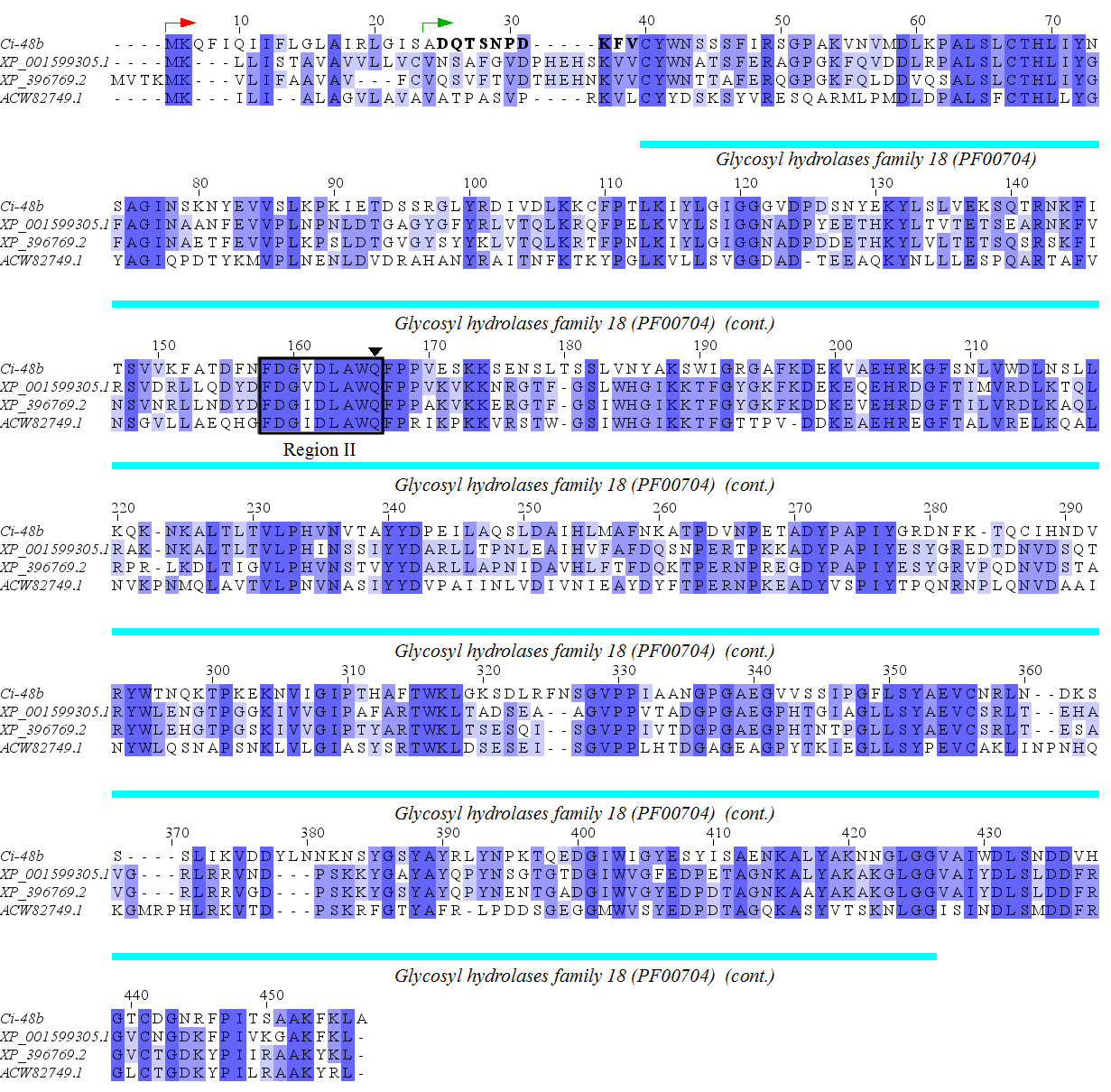

Supplement: Additional file 4 — Amino acid sequence alignment of Imaginal disc Growth Factors (IDGFs)-like proteins from different insect species. The sequence of the Ci-48b from C. inanitus was aligned with sequences from the following species: N. vitripennis [GenBank:XP_001599305.1], A. mellifera [GenBank:XP_396769.2] and Manduca sexta [GenBank:ACW82749.1]. The conserved region II is boxed. Triangle indicates a glutamine residue replacing, in these proteins, a glutamic acid residue of functional importance. Location of the glycosyl hydrolase family 18 domain is indicated by a blue line. Red and green arrows indicate the beginning of the predicted signal peptide and mature protein sequences of Ci-48b, respectively. Sequence printed in bold was also obtained by N-terminal sequencing of the Ci-48b protein. [file 1471-2164-11-693-S4.PNG]

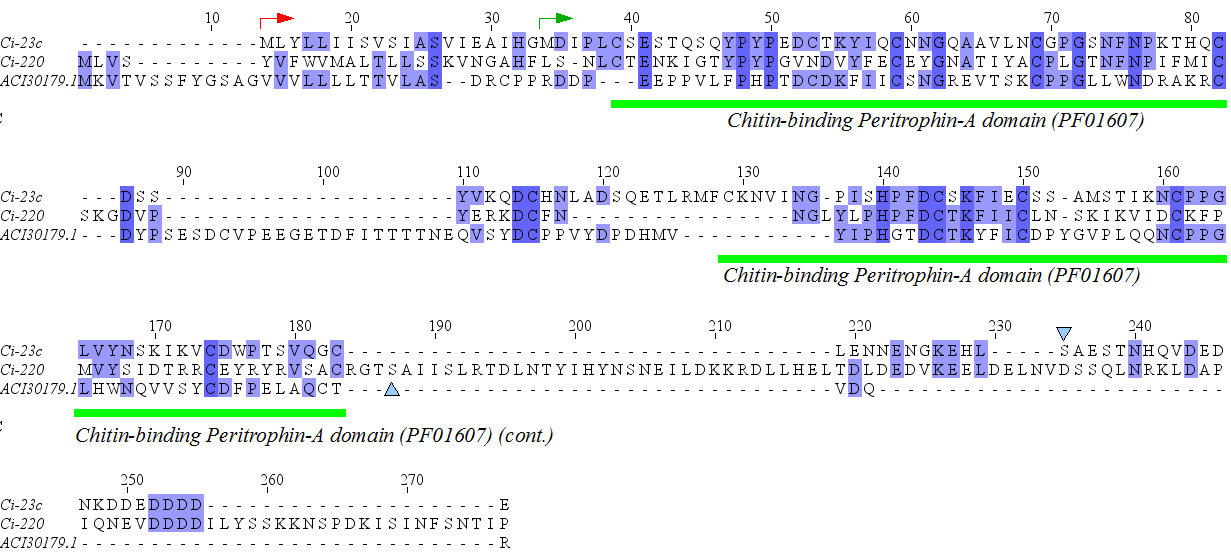

Supplement: Additional file 5 — Amino acid sequence alignment of Ci-23c, Ci-220 and AD-873. The sequences of the Ci-23c and Ci-220 proteins from C. inanitus were aligned with the sequence of the AD-873 protein from Anopheles darlingi [GenBank:ACI30179.1]. Location of the chitin-binding Peritrophin-A (CBM_14) domains of Ci-23c are indicated by green lines under the alignment. Serine residues that potentially serve as glycosaminoglycan attachment sites are indicated by blue triangles. Red and green arrows indicate the beginning of the predicted signal peptide and mature protein sequences of Ci-23c, respectively. [file 1471-2164-11-693-S5.PNG]

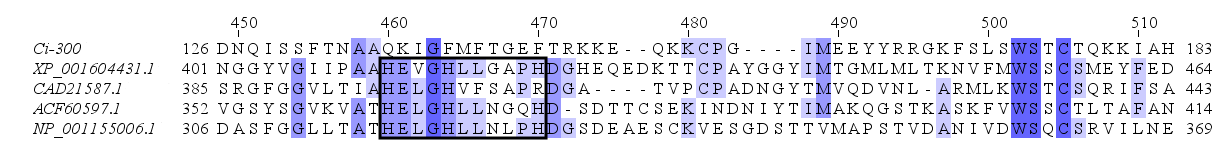

Supplement: Additional file 6 — Partial amino acid sequence alignment of Ci-300 with insect metalloproteases. The sequence of Ci-300 was aligned with sequences of metalloproteases from the following species: N. vitripennis [NCBI Reference Sequence:XP_001604431.1] and [NCBI Reference Sequence: NP_001155006.1], P. hypochondriaca [GenBank:CAD21587.1] and E. pennicornis [GenBank:ACF60597.1]. The Zn2+-binding motif of HExxHxxGxxH featuring known metalloproteases' partial alignment is boxed in black. [file 1471-2164-11-693-S6.PNG]

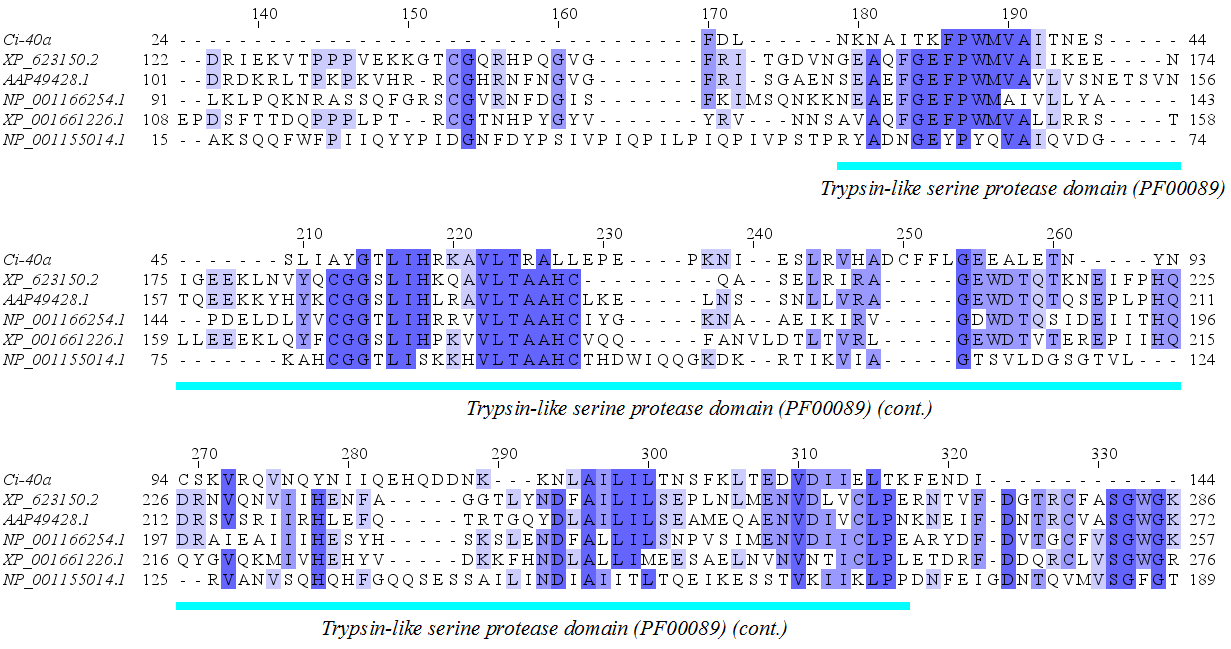

Supplement: Additional file 7 — Amino acid sequence alignment of Ci-40a with serine protease homologs (SPHs). The partial sequence of Ci-40a was aligned with sequences of SPHs from the following species: A. mellifera [NCBI Reference Sequence:XP_623150.2], C. rubecula [GenBank:AAP49428.1], N. vitripennis [NCBI Reference Sequence:NP_001166254.1] and [NCBI Reference Sequence:NP_001155014.1] and A. aegypti [NCBI Reference Sequence:XP_001661226.1]. The location of the trypsin-like serine protease domain of Ci40a is indicated by a blue line under the alignment. [file 1471-2164-11-693-S7.PNG]

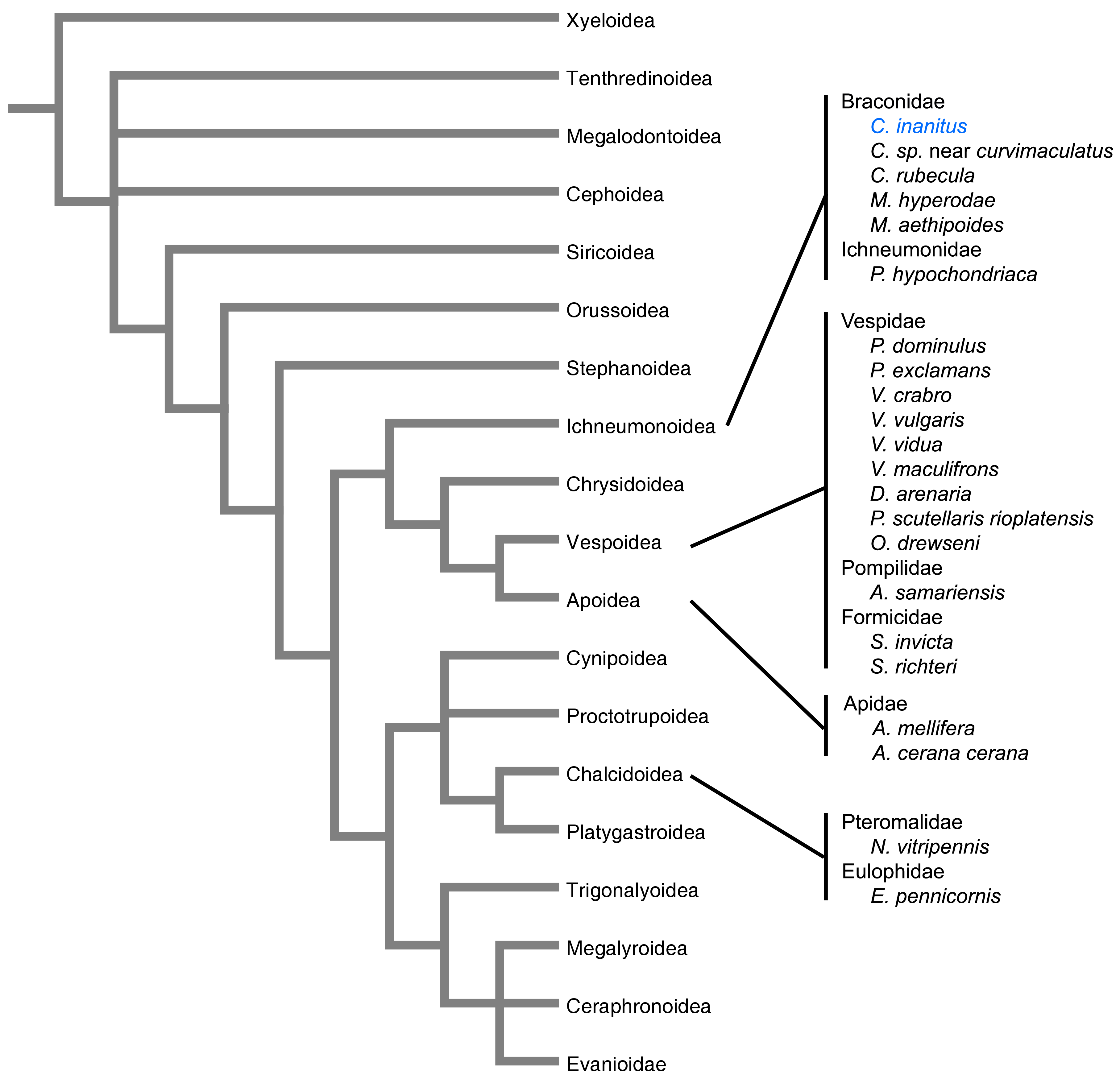

Supplement: Additional file 9 — Phylogeny of the major superfamilies of Hymenoptera. Family and species names discussed in the present paper are indicated on the right side of the figure. The phylogeny of Hymenoptera shown on the left side of the figure is adapted from [9]. [file 1471-2164-11-693-S9.PNG]
